# Dissociation Does Not Mediate Antidepressant Response to Esketamine: A Real-World Longitudinal Study with 6-Month Follow-Up

**DOI:** 10.1192/j.eurpsy.2026.10595

**Published:** 2026-07-31

**Authors:** M. Olivola, V. Martiadis, K. La Monica, C. M. Angeletti, A. Frediani, V. Casati, R. Anniverno, G. Versaci, T. Prodi, N. Brondino, B. M. Dell’Osso

**Affiliations:** 1 Department of Biomedical and Clinical Sciences Luigi Sacco, Department of Psychiatry; 2 Ospedale Macedonio Melloni, ASST FBF SACCO, MILAN; 3 Department of Mental Health, Asl Napoli 1 Centro, naples; 4 Department of Biomedical and Clinical Sciences Luigi Sacco, Department of Psychiatry, ASST; 5 Department of Biomedical and Clinical Sciences Luigi Sacco, Department of Psychiatry, Fatebenefratelli-Sacco, University of Milan; 6 Department of Biomedical and Clinical Sciences Luigi Sacco, Department of Psychiatry, ASST, ASST FBF SACCO, MILAN; 7 Department of Brain and behavioural sciencel, università di pavia, Pavia, Italy

## Abstract

**Introduction:**

The role of dissociation in mediating antidepressant response to intranasal esketamine remains debated. While dissociative experiences are often observed during treatment, their predictive value for clinical outcomes is uncertain.

**Objectives:**

We aimed to assess whether (i) baseline dissociative traits (DES-II) and (ii) early changes in dissociation (ΔDES T0→T2) predict short-term antidepressant response, and to explore whether dissociation mediates depressive symptom improvement across 6 months of follow-up.

**Methods:**

This multicenter naturalistic cohort included 31 patients with treatment-resistant depression (mean age 44.7±11.6) treated at academic community clinics in Pavia and Milan. Clinical assessments included MADRS at baseline (T0), 2 weeks (T1), 1 month (T2), 2 months (T3), 3 months (T4), and 6 months (T5) (Montgomery & Åsberg, Br J Psychiatry 1979). Dissociation was measured with DES-II at T0 and T2 (Carlson & Putnam, Dissociation 1993). Outcomes: % MADRS change and responder status (≥50% reduction). Predictors: DES-II at baseline and ΔDES T0→T2. Covariates: baseline MADRS, age, RMET (Baron-Cohen et al., J Child Psychol Psychiatry 2001), Psychache . Analyses included linear/logistic regressions and exploratory mediation (Preacher & Hayes, Behav Res Methods 2008).

**Results:**

Mean MADRS decreased from 31.9±7.9 to 16.7±9.6 at T2 (−47%, responders 48%). Baseline DES-II (mean 0.185±0.12) showed no independent association with response (OR 0.18; 95%CI 0.00–15.5; p = 0.38). ΔDES T0→T2 did not predict response (OR 0.01; 95%CI 0.00–80.5; p = 0.36) nor % improvement (β = 12.7; p = 0.33). Mediation analysis revealed no significant indirect effect (a*b ≈ 10.7%; 95%CI −21.7–79.9). By DES tertiles, % improvement was comparable (Low 42.6%; Mid 51.0%; High 46.5%). Longitudinal follow-up (T1–T5) confirmed sustained MADRS reduction independent of dissociation. (Figure 1 Madrs reduction across timepoints, Figure 2 %madrs improvement by baseline DES, Figure 3 mediation model)

**Image 1:**
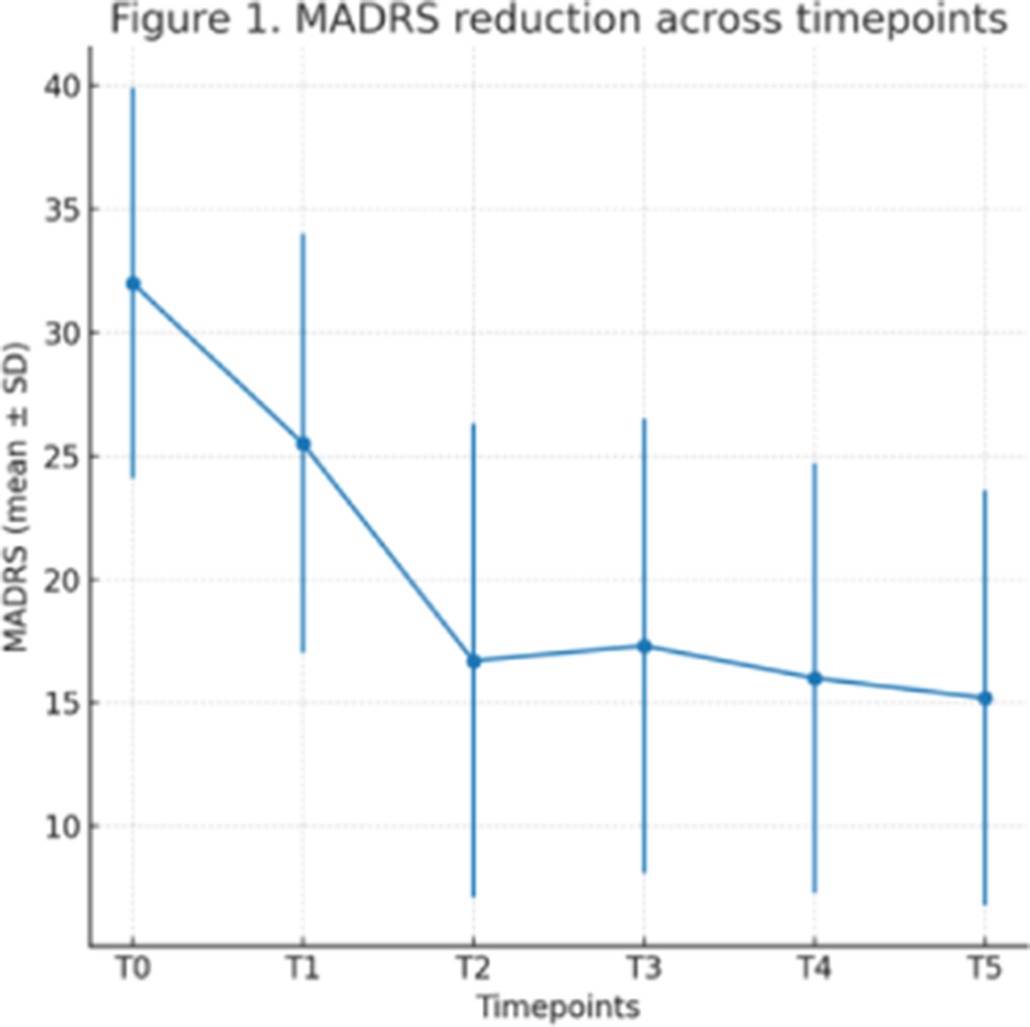
Image 1: Long description.

**Image 2:**
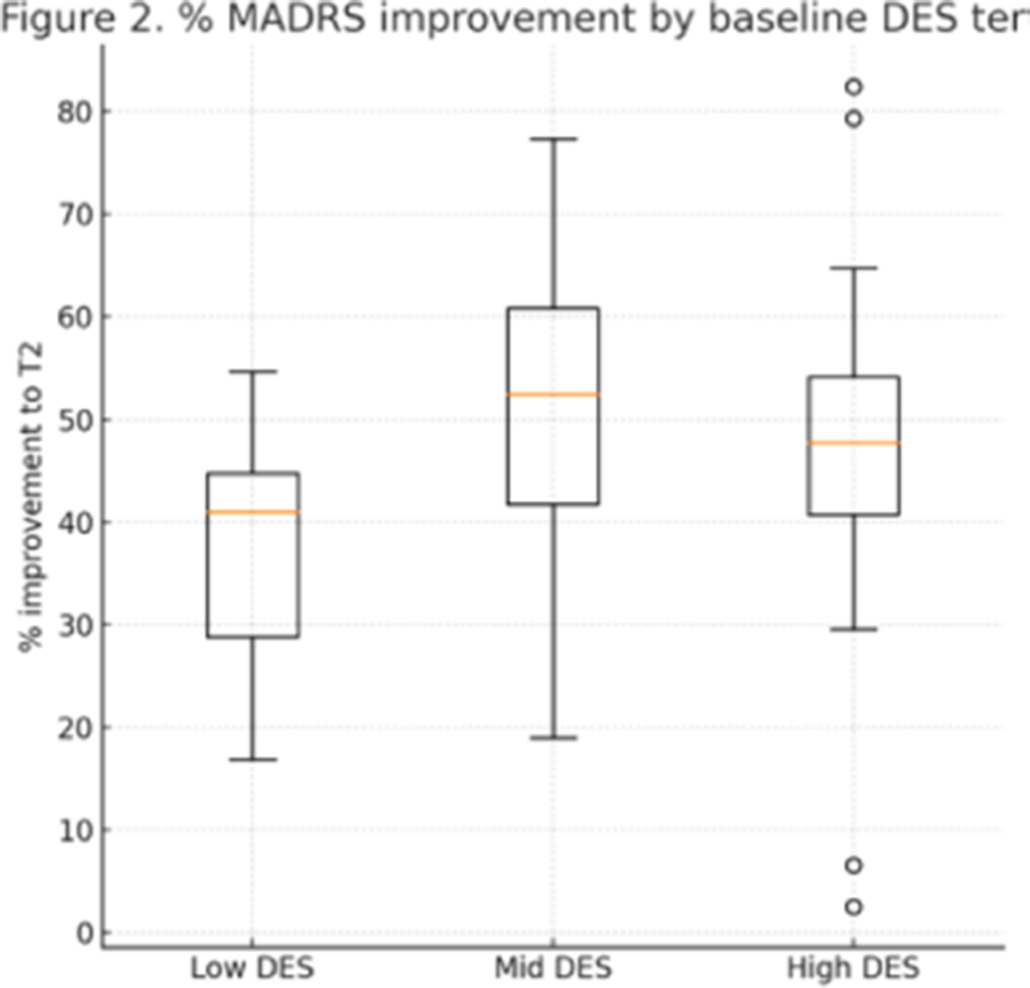
Image 2: Long description.

**Image 3:**
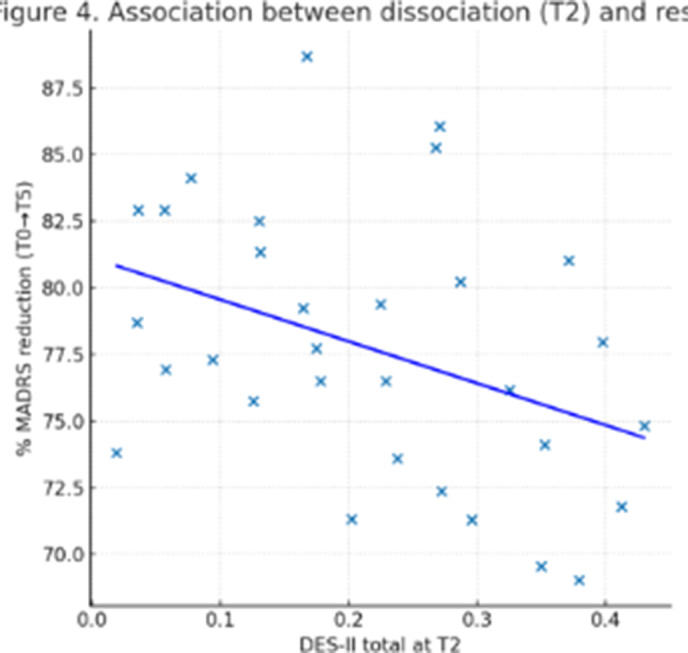
Image 3: Long description.

**Conclusions:**

In this real-world cohort, dissociative traits and early changes in dissociation did not predict or mediate antidepressant response to intranasal esketamine. However, in clinical practice patients frequently report intense dissociative experiences, and psychotherapists describe esketamine as facilitating emotional processing of unelaborated traumatic memories (DeCou et al., Behav Ther 2019). This suggests that standard trait-based measures such as DES-II may introduce bias and fail to capture clinically meaningful dissociative phenomena. Future studies should employ session-level state measures (e.g., CADSS, ASC-5), cumulative dosing parameters, and longitudinal mixed-effects models to disentangle trait versus state contributions and clarify the therapeutic relevance of dissociation.

**Disclosure of Interest:**

None Declared

## Long descriptions

### Image 1: Long description

The Y-axis is labeled M A D R S (mean plus or minus S D) with a scale from 10 to 40. The X-axis is labeled Timepoints with six categorical markers: T 0, T 1, T 2, T 3, T 4, and T 5.

* At T 0, the mean score is approximately 32 with a large vertical error bar extending from 24 to 40.

* At T 1, the score drops to approximately 25.5.

* At T 2, there is a sharp decrease to approximately 16.5.

* At T 3, the score plateaus with a slight increase to approximately 17.5.

* At T 4, the score decreases slightly to 16.

* At T 5, the score reaches its lowest point at approximately 15.

The overall trend shows a significant initial reduction in M A D R S scores between T 0 and T 2, followed by a stabilization or very gradual decline through T 5. Vertical error bars representing standard deviation are present at every data point.

Navigate back to Image 1:.

### Image 2: Long description

The y-axis is labeled percent improvement to T 2, ranging from 0 to 80 in increments of 10. The x-axis contains three categories: Low D E S, Mid D E S, and High D E S.

* Low D E S: The median improvement is approximately 41 percent. The interquartile range spans from roughly 29 to 45 percent, with whiskers extending from 17 to 55 percent.

* Mid D E S: The median improvement is higher at approximately 53 percent. The interquartile range spans from 42 to 61 percent, with whiskers extending from 19 to 77 percent.

* High D E S: The median improvement is approximately 48 percent. The interquartile range spans from 41 to 54 percent, with whiskers extending from 30 to 65 percent. This group includes four outliers: two high outliers near 80 and 83 percent, and two low outliers near 3 and 7 percent.

Navigate back to Image 2:.

### Image 3: Long description

The X-axis is labeled D E S dash I I total at T 2 with values ranging from 0.0 to 0.4. The Y-axis is labeled percentage M A D R S reduction (T 0 arrow T 5) with values ranging from 70.0 to 87.5.

* A solid blue regression line starts at approximately 81 percent on the Y-axis and follows a linear decrease to approximately 74 percent at the far right of the X-axis.

* Individual data points are represented by blue x marks scattered across the plot area.

* The highest concentration of data points is between 0.1 and 0.3 on the X-axis and 72.5 and 82.5 on the Y-axis.

* Outliers include a point near the top center at approximately (0.17, 88.5) and several points in the lower right quadrant near (0.38, 69.0).

Navigate back to Image 3:.

